# Intermittent fasting combined with resistance training: effects on body composition, muscular performance, and dietary intake

**DOI:** 10.1186/1550-2783-12-S1-P38

**Published:** 2015-09-21

**Authors:** Grant M Tinsley, Natalie K Butler, Jeffrey S Forsse, Annie A Bane, Grant B Morgan, Paul S Hwang, Peter W Grandjean, Paul M La Bounty

**Affiliations:** 1 Baylor University, Waco, TX, USA

## Background

Intermittent fasting (IF) is a dietary strategy that has recently gained popularity due to a number of potential health benefits. One form of IF, termed time-restricted feeding (TRF), only allows caloric intake during a limited window of time each day (often 4 to 8 hours in duration). One concern of IF is the potential loss of lean mass due to the fasting periods. Resistance training is known to help mitigate loss of lean mass during hypocaloric diets. The purpose of this experiment was to examine the effects of TRF in combination with resistance training on body composition, muscular performance, and dietary intake in young untrained males.

## Methods

Adult males (n = 18) were recruited and randomized into one of two groups: resistance training alone (RT) or resistance training plus TRF (RT+TRF). Both groups followed a 3-days-per-week resistance training program for 8 weeks. The TRF program was implemented on non-workout days (i.e. 4 days per week) and consisted of consuming all calories within any 4-hour period between 4 PM and midnight. Both groups were allowed unrestricted food intake during feeding periods. Research visits were conducted at baseline, 4 weeks, and 8 weeks after beginning the study and consisted of body composition assessment via dual-energy x-ray absorptiometry (DXA), 1-repetition maximum (1-RM) strength testing and muscular endurance testing on bench press and leg press exercises, and subjective measures of program difficulty. Diet records, workout logs, and compliance forms were used to track and encourage program adherence, as well as examine dietary differences. One-way and factorial ANOVAs were conducted using R (version 3.1.1).

## Results

No group*time interactions were found for any measures of body composition (lean mass, fat mass, and body fat percentage), muscular performance, or dietary intake. A time main effect for increased leg press 1-RM (p = 0.011) and a group main effect for higher leg press 1-RM in the RT+TRF group (p = 0.011) were seen. A group main effect was present for higher bench press endurance in the RT+TRF group (p = 0.013). Within the RT+TRF group, participants consumed fewer calories (p = 0.008), less protein (p = 0.017), less carbohydrate (p = 0.007), and less fat (p = 0.050) on fasting days compared to non-fasting days. However, there was no difference in the percent of total calories from any macronutrient. There were no differences in total calories, protein, or fat consumed between the non-fasting days of the RT+TRF group and the RT group, but the RT group consumed more carbohydrate (p = 0.018). Noticeable differences in individual responses to the programs were noted.

## Conclusions

In the absence of any other dietary guidance, restricting caloric consumption to a 4-hour window on 4 days per week was not sufficient to elicit body composition improvements in 8 weeks, although lean mass was maintained in both groups. This form of IF was sufficient to reduce caloric intake on fasting days, but this did not translate to body fat reductions in many subjects. Untrained young men experience similar strength adaptations whether they eat normally or perform this form of IF. Protein intake may be of particular concern for individuals implementing IF and young men beginning a resistance training program.

